# Assessment and management of anxiety disorders in children and adolescents

**DOI:** 10.1136/archdischild-2013-303768

**Published:** 2014-03-17

**Authors:** Cathy Creswell, Polly Waite, Peter J Cooper

**Affiliations:** 1 School of Psychology and Clinical Language Sciences, University of Reading, Reading, Berkshire, UK; 2 Department of Psychology, Stellenbosch University, Matieland, South Africa

**Keywords:** Anxiety, Assessment, Treatment, Children, Adolescents

## Abstract

Anxiety disorders in childhood and adolescence are extremely common and are often associated with lifelong psychiatric disturbance. Consistent with DSM-5 and the extant literature, this review concerns the assessment and treatment of specific phobias, separation anxiety disorder, generalised anxiety disorder, social anxiety disorder, panic disorder and agoraphobia. Evidence-based psychological treatments (cognitive behaviour therapy; CBT) for these disorders have been developed and investigated, and in recent years promising low-intensity versions of CBT interventions have been proposed that offer a means to increase access to evidence-based treatments. There is some evidence of effectiveness of pharmacological treatments for anxiety disorders in children and young people, however, routine prescription is not recommended due to concerns about potential harm.

## Introduction

Anxiety disorders are among the most common psychiatric conditions in young people, with community studies indicating a period prevalence between 9% and 32% during childhood and adolescence.^1^ They typically have an adverse impact on educational achievement, family life and leisure activities^2^ ^3^; and they often co-occur with other anxiety disorders, depression and behavioural disorders.^4^ Anxiety disorders in young people are associated with increased rates of anxiety and depression in early adulthood, as well as with a number of other adverse mental health and life course outcomes.^5^ ^6^ Indeed, for the majority of adults with anxiety disorders and depression the onset of psychological difficulties was in childhood or adolescence, with anxiety disorders being the most common prior diagnosis.^7^ Despite the significant public health burden associated with anxiety disorders in children and young people, they commonly remain untreated.^8^ Collectively these considerations highlight the importance of early access to effective identification and treatment.

### Classification of anxiety disorders among children and adolescents

There have been some recent changes to the classification of anxiety disorders. Consistent with the previous version of the Diagnostic and Statistical Manual of Mental Disorders (DSM-IV^9^), DSM-5^10^ includes the following anxiety disorders: specific phobia, generalised anxiety disorder, social anxiety disorder (formerly social phobia), panic disorder and agoraphobia. Key changes in DSM-5 include: (i) agoraphobia has been classified as a stand-alone diagnosis (ie, no longer linked to the presence or absence of panic disorder), (ii) separation anxiety disorder and selective mutism have been re-classified as anxiety disorders (rather than in a section for ‘disorders usually first diagnosed in infancy, childhood or adolescence’) and (iii) obsessive-compulsive disorder, post-traumatic stress disorder and acute stress disorder are, respectively, grouped under obsessive-compulsive and related disorders, and trauma-related and stressor-related disorders (ie, no longer included within the anxiety disorders category).^i^ While core features of each anxiety disorder are broadly consistent with DSM-IV, in order to minimise the over-diagnosis of transient fears for agoraphobia, specific phobia and social anxiety disorder, those under the age of 18 are now required to have had symptoms for at least 6 months.

## Assessment

Young people with anxiety disorders are unlikely to present for help independently, with parents commonly raising concerns to general practitioners. The challenge in assessing for the presence of anxiety disorders is distinguishing pathology from ‘normal’ developmentally appropriate fears and worries. As anxiety disorders represent an extreme presentation of normal events, this distinction is essentially made on the basis of the severity and persistence of symptoms and the degree of associated impairment. Structured interview schedules used for assessing the presence of anxiety disorders typically establish whether a child meets symptom criteria for a specific anxiety diagnosis, as well as the degree to which these symptoms interfere with functioning. The most widely used diagnostic schedule is the Anxiety Disorders Interview Schedule for children and parents (ADIS-C/P^18^) which follows DSM-IV criteria. Questionnaire measures with normative data also provide a useful indication of whether symptoms are present at a clinical level. While there are no brief screening measures for use with children and young people, parent and child report measures such as the Revised Children's Anxiety and Depression Scale (RCADS^19^) and the Spence Children's Anxiety Scale (SCAS^20^) contain scales that broadly align with diagnostic categories. These tools are valuable in helping to identify clinical levels of anxiety among young people (as diagnostic cut-offs are available) and they can be useful in monitoring progress through treatment. Although National Institute of Health and Care Excellence (NICE) guidelines relating to anxiety disorders principally relate to adults, the recent NICE guideline for the assessment and treatment of social anxiety disorder^21^ highlights a number of considerations for assessment with young people which can be applied across the anxiety disorders. It is noted, in particular, that once potential concerns relating to anxiety have been identified in a young person, a comprehensive assessment should be conducted by an appropriate healthcare professional. This should include an opportunity for interviewing the young person on their own; and it should also involve interviewing a parent, carer or other adult who knows the child well and can report on current and past behaviour. Since there is a high level of comorbidity in young people with anxiety disorders, it is essential to assess for possible co-existing mental health problems, neurodevelopmental conditions, drug and alcohol misuse, and speech and language problems.

## Management

The most commonly evaluated treatments for anxiety disorders in children and adolescence are psychological approaches, especially cognitive behaviour therapy (CBT). One of the first manualised CBT programmes for the treatment of anxiety disorders in young people was Kendall's ‘Coping Cat’.^22^ This includes components concerned with psycho-education, identification and modification of negative automatic thoughts, exposure to feared stimuli, problem solving, and training in coping skills. Most programmes subsequently developed are heavily influenced by ‘Coping Cat’.

The great majority of young people with anxiety disorders do not access clinical services.^23^ This is likely to be the result of a lack of awareness and knowledge, lack of appropriate local services, long waiting lists for treatment, competing family time commitments and a paucity of trained professionals. In line with guidance for increasing access to treatment in adult populations,^24^ recent therapeutic innovations have focused on brief or low-intensity versions of CBT that can be delivered by non-specialists, with the ultimate aim of delivering treatment within a stepped-care framework. The logic here is to deliver brief, relatively simple, first-line treatments routinely to service users with a relatively good prognosis, reserving more intensive treatments for those who do not respond to the first-line treatment and those whose prognostic profile indicates that they require more input.^25^

### Low-intensity interventions for anxiety disorders in childhood and adolescence

Two low-intensity approaches have been subjected to systematic evaluation: (i) brief treatments in which parents are guided to work through a book that instructs them on how to help their child overcome their difficulties with anxiety (‘bibliotherapy’) and (ii) treatments delivered via computerised platforms (‘e-therapies’).

#### Bibliotherapy

Promising evidence is emerging of impressive clinical gains using low-intensity CBT for child anxiety delivered by parents. Notably, in an Australian study, the simple provision of a workbook for parents was associated with recovery in 26% of anxious children aged 6–12 years, compared to 7% who received no treatment.^26^ The addition of therapist support, however, has been found to be associated with substantially higher recovery rates. Lyneham and Rapee,^27^ for example, in a study of the treatment of childhood anxiety in a rural Australian population, supplemented a workbook for parents with nine scheduled weekly phone calls or emails. Almost 90% of children no longer met diagnostic criteria for their primary anxiety disorder following treatment, and gains were maintained at a 12-month follow-up. Notably, in a further Australian study of the treatment of anxious children, no difference in child outcome was found between bibliotherapy delivered via parents (supported by a 2-h group plus regular phone calls) compared to outcome following twelve 60–90 min sessions of CBT delivered with parents and children.^28^ A similar approach has recently been applied with a referred population from a UK National Health Service setting.^29^ A sample of anxious children (of non-anxious mothers) received an intervention where parents were given a CBT manual^30^ and 5 h 20 min contact (face-to-face sessions and telephone reviews) with a therapist to help them apply the manual's principles. Immediately following the intervention 50% of children were free of their primary diagnosis (double the rate in the waitlist control group), increasing to over 70% at 6-month follow-up. Notably, in this study, level of therapist experience was unrelated to child outcome, suggesting that this low-intensity approach can be delivered effectively by novice therapists. Together these studies suggest that therapist guided bibliotherapy, delivered via parents, is an effective and efficient treatment for child anxiety.

#### E-therapies

Two computerised treatment programmes for childhood anxiety disorders have been developed and systematically evaluated: BRAVE for Children-Online^31^ and Camp-Cope-A-Lot: The Coping Cat.^32^ Both programmes involve the child completing 10–12 computerised CBT sessions, with some additional sessions for parents. Sessions are therapist-supported. In BRAVE for Children-Online, the therapist provides weekly personalised emails and one telephone call to help the child plan their exposure hierarchy. In Camp-Cope-A-Lot, the therapist is present for six sessions and assists in implementing exposure tasks. In studies of both approaches computerised treatment was superior to the waitlist and received high patient satisfaction ratings. March *et al*^31^ found that 30% of the children who received BRAVE for Children-Online were free of their primary diagnosis immediately post-treatment; however, few children had completed all therapy sessions. By 6-month follow-up, when 62% of children had completed all sessions, 75% of recruited participants were free of their primary diagnosis. Khanna and Kendall^32^ found Camp-Cope-A-Lot to be as effective as face-to-face CBT; 81% were free of their primary diagnosis at post-treatment with progress maintained at 3-month follow-up.

Three computerised treatments designed specifically for anxious adolescents have been evaluated: BRAVE for Teenagers-Online,^33^ Cool Teens^34^ and Think, Feel Do.^35^ Spence *et al*^33^ compared BRAVE for Teenagers-Online to individual, clinic-based CBT and found no differences between groups post-treatment and at follow-up assessments. By the 12-month follow-up, 68% of those who received the online treatment were free of their primary anxiety disorder diagnosis. In Cool Teens, the adolescent undertakes 12 sessions via a CD-ROM, with parent support and regular telephone calls from a therapist. Wuthrich *et al*^34^ compared Cool Teens with a waitlist and found similar end of treatment results to the Spence *et al*^33^; however, in contrast to Spence, there was deterioration by the 3-month follow-up. Finally, encouraging preliminary findings have been obtained using the Think, Feel, Do programme in relation to symptoms of social anxiety.^35^ Taken together, these studies suggest that e-therapies may be a promising low-intensity approach for the treatment of anxiety disorders in children and adolescents.

### High-intensity interventions for anxiety disorders in childhood and adolescence

More intensive treatments for childhood anxiety, typically involving 9–20 face-to-face CBT treatment sessions, have been subjected to extensive evaluation. In a Cochrane review, James *et al*^36^ identified 41 systematic evaluations of CBT with children and young people with anxiety disorders aged above four and below 19 years of age. The odds ratio (OR) for remission across these studies was 0.13 for CBT versus waiting list controls, with CBT effective in over 59% of cases compared to 18.4% in controls. Method of treatment delivery (ie, individually with the child, in a group, or with parents/the family) was not systematically related to outcome. Notably, eight of these studies included an active therapeutic comparison condition (eg, educational support) and, collectively, these studies did not show superior outcomes for those receiving CBT. While one could not, therefore, conclude from the current evidence base that CBT is the *most* effective form of treatment, it is the only treatment with an adequate evidence base to support its general application.

The majority of randomised controlled trials to evaluate CBT for anxiety disorders in young people have included children over 7 years of age. The extent to which CBT is applicable to younger children has been debated. It has been questioned whether young children have the cognitive capacities to benefit from the cognitive components of treatment; and it has been noted that the modification of young children's behaviour is heavily reliant on parental influences. Nevertheless, some positive findings have been reported with young children. Hirshfeld-Becker *et al*^37^ demonstrated the effectiveness of a parent and child programme for the treatment of 4–7-year-old children with an anxiety disorder, as did Waters, Ford, Wharton and Cobham^38^ with 4–8-year-old children. In the latter study similar outcomes were found whether the treatment was delivered with parents and children or with parents alone. A group CBT programme delivered solely to parents has also been shown to be effective for 2–9-year-old children with anxiety disorders.^39^

The studies described above have typically included children with a range of anxiety disorders. This is partly driven by theoretical considerations, in that there are no well-validated diagnosis-specific models of the maintenance of disorder in children and young people; and partly for practical reasons, in that there is such a high level of comorbidity between the anxiety disorders.^40^ There has been some recent suggestion, however, that disorder-specific treatments may be associated with better outcomes for some disorders, especially social anxiety disorder. Indeed, a number of recent reports have noted that children and young people with social anxiety disorder do not make as substantial gains from generic treatment as those with other anxiety disorders.^41–43^ Correspondingly, recent meta-analyses have revealed better outcomes for young people with social anxiety disorder following disorder-specific compared to generic treatments.^11^ ^44^ These ‘specific’ treatments have typically included the addition to generic treatments of social skills training and opportunities for peer interactions. There has been some interest in specific treatments for other conditions, such as specific phobias^45^ and separation anxiety disorder.^46^ For separation anxiety disorder no clear advantage over generic treatments has emerged to date.^46^ For specific phobias, direct comparisons with generic treatments have not been conducted; however, some evidence suggests that taking an especially focused approach may allow for particularly rapid treatment. Thus, brief exposure-based treatment has been found to be effective compared to an active (education support) control, with 55% of children being free of their specific phobia after a single treatment session.^45^

### Considerations for good practice in the delivery of psychological interventions for children and young people with anxiety disorders

The recent NICE guidelines for the assessment and treatment of social anxiety disorder^21^ set out treatment principles for working with children and young people that apply well across the anxiety disorders. Specifically, it is noted that all interventions should be delivered by competent practitioners who receive regular, high-quality supervision that is informed by routine monitoring of treatment progress from each treatment session. The content of treatment should be based on relevant evidence-based treatment manuals, and adherence to these manuals should be monitored and evaluated in supervision, for example, using video or audio recordings of treatment sessions. Therapists should be aware of the potential impact of the child's home, school and wider social environment on the maintenance and treatment of anxiety disorders, so should work with the child or young person's parents, teachers or peers, as appropriate, to create an environment that supports the goals of treatment.

### Medication

There is some evidence that medication can be effective in treating anxiety in children and adolescents, at least in the short term. A recent meta-analysis^47^ showed anxiolytic medication to be associated with a significantly greater clinical response than a placebo drug (58.1% vs 31.5%). Selective serotonin reuptake inhibitors (SSRIs) are regarded as the pharmacological treatment of choice for anxiety disorders in children and adolescents because of their effectiveness and safety profile. It is important to note that benzodiazepines have not been systematically evaluated in children and adolescents and, in view of concerns about dependency and side effects,^48^ their use is not recommended.^21^

The relative effectiveness of psychological and pharmacological treatments for anxiety disorders in children and young people has been assessed in two trials. Beidel *et al*^49^ compared SSRIs to CBT in the treatment of social anxiety disorder in children and adolescents (7–17 years) and found CBT to be more effective than fluoxetine at the end of the treatment phase; however, Walkup *et al*^50^ in a sample of children and young people with a range of anxiety disorders, found no difference in outcome immediately after treatment between those treated with sertraline and those treated with CBT. Walkup and colleagues did, however, find the combination of sertraline and CBT to be more effective than either treatment alone. In the latter study, by 24 weeks after the end of acute treatment (with ongoing maintenance of sertraline or CBT booster sessions), there continued to be no significant differences between CBT and sertraline, although the advantage of the combined treatment had attenuated somewhat.^51^

These recent findings suggest that CBT, sertraline and their combination are all possible options for the treatment of childhood anxiety disorders. However, several important questions concerning the use of anxiolytic medication in children remain unanswered. Thus, it is unclear if there is an age below which medication would be contraindicated; it is uncertain what the duration of treatment should be; and, most notably, it is unknown what the clinical consequences are of stopping a course of medication.^48^ As a result of these uncertainties, together with parental preference for psychological over pharmacological interventions for their children,^52^ the NICE guidelines for social anxiety disorder^21^ recommend that medication should not be routinely offered to children and young people and that psychological treatment should be considered the first-line treatment. Although NICE is yet to report on the non-social forms of anxiety disorders in childhood and adolescence, there is nothing in the literature to suggest that their conclusion will be different concerning the use of anxiolytic medication in these other forms of anxiety disorder.

## Future directions for clinical research

There is now convincing evidence that with good psychological treatment the majority of children and young people with anxiety disorders will show favourable outcomes. Little is known, however, about why treatment is sometimes ineffective. Recently several independent predictors of treatment outcome from CBT have been identified: gender, primary anxiety severity, comorbid mood and externalising disorder, and the genetic factors 5*HTTLPR, NGF* rs6330.^53^ The clinical utility of these predictors remains to be determined. Notably, studies to date have been limited in terms of consideration of ethnicity and culture, and the extent to which treatment applies across cultures remains to be established. Further, the extent to which treatments apply to those with comorbid conditions is also yet to be firmly established. James *et al*^36^ identified six studies of the treatment of anxiety disorder among children and young people with autistic spectrum disorders and found significant reductions in anxiety diagnoses compared to a waitlist control condition. The interventions were typically modified to include more applied behavioural analysis and greater repetition of material over more sessions. These studies are encouraging in demonstrating that, with appropriate adaptation, existing treatments can be effectively applied to broader populations that those typically included in treatment trials. Finally, further research is required to determine whether children and young people who do not respond to CBT may benefit from subsequent pharmacotherapy, in the short and long term.

## Summary and conclusions

Anxiety disorders in childhood and adolescence are common, often stable and present a risk for lifelong psychiatric disturbance. It is a cause for concern that, despite the existence of evidence-based interventions, the majority of children and young people with anxiety disorders do not access treatment. Numerous trials have now supported the effectiveness of CBT for childhood anxiety disorders, compared to a waitlist condition. In recent years, a number of promising low-intensity CBT-based treatments have been developed and evaluated that offer a mechanism for increasing access to evidence-based treatment for children and young people with anxiety disorders. While there is some evidence for the effectiveness of SSRI medication, this is to be weighed up against the potential risk of harm in this age group, and the fact that so many questions concerning administration remain unanswered. In view of these concerns, recent guidelines state that pharmacological treatments should not be offered routinely to children and young people.

## Footnotes

i Consistent with the reclassification in DSM-5, we have not included OCD and PTSD in this review. In any event, treatment research for these disorders has, in the main, involved the assessment of disorder-specific treatments^11^ and clinical recommendations for these two conditions are, therefore, somewhat different from those for disorders under consideration in this paper.^12^ ^13^ We have also not included selective mutism although this has now been classified as an anxiety disorder within DSM-5. There has been little systematic evaluation of treatments beyond uncontrolled case studies and single-participant experiments.^14^ Two small controlled trials using fluoxetine^15^ ^16^ indicate that it may be of some benefit and more recently, a small randomized controlled pilot study has provided preliminary evidence for the effectiveness of behaviour therapy.^17^ Consequently as treatment design and evaluation for selective mutism is at such an early stage, and because children with selective mutism have not typically been included in treatments for general anxiety disorders, it is not included within the body of this review.
